# Supplementary material for: Osteoprotegerin-dependent M cell self-regulation balances gut infection and immunity
Source: Nat Commun. 2020 Jan 13;11:234. doi: 10.1038/s41467-019-13883-y (PMC6957684; doi:10.1038/s41467-019-13883-y)
Supplement: Supplementary file 1 — Supplementary Information [file 41467_2019_13883_MOESM1_ESM.pdf]

Supplementary Information

Osteoprotegerin-dependent M-cell self-regulation balances gut infection and immunity

Kimura et al.

Supplementary Figure 1

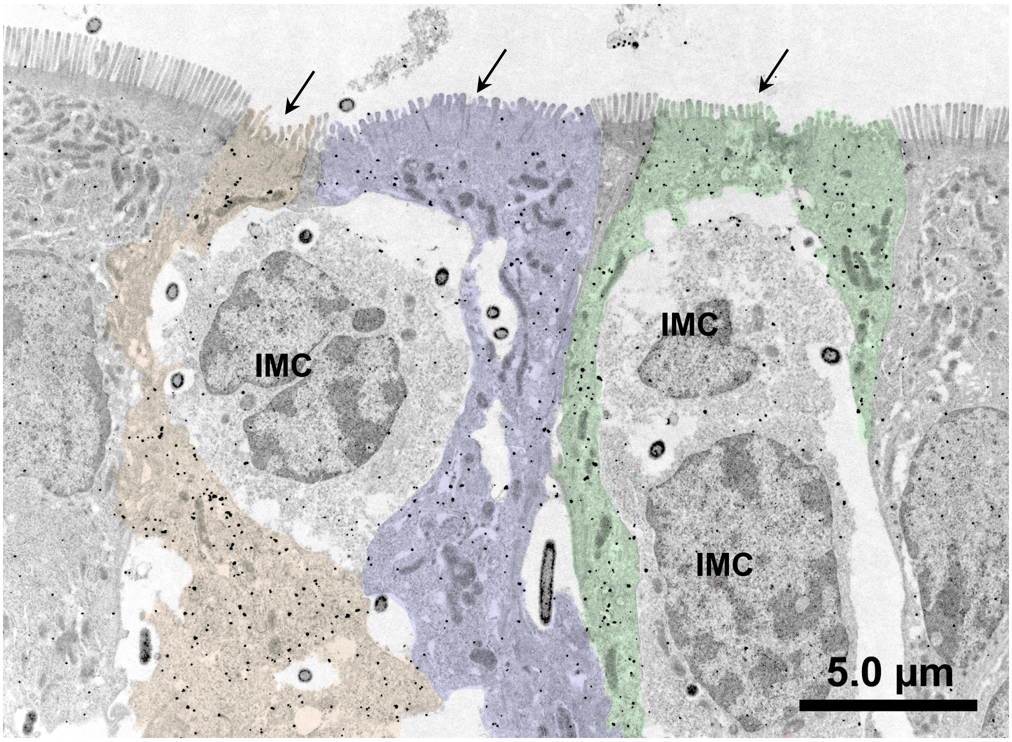

Supplementary Figure 1
Transmission electron micrographs show OPG immunostaining within FAE.
Immunoreactivities of OPG (black dots) were detected predominantly in M cells (colored orange, blue, and green) that lack a brush border composed of long microvilli at its apical surface (arrows) and embrace intraepithelial migrating cells (IMCs). Scale bar: 5 µm.

Supplementary Figure 2

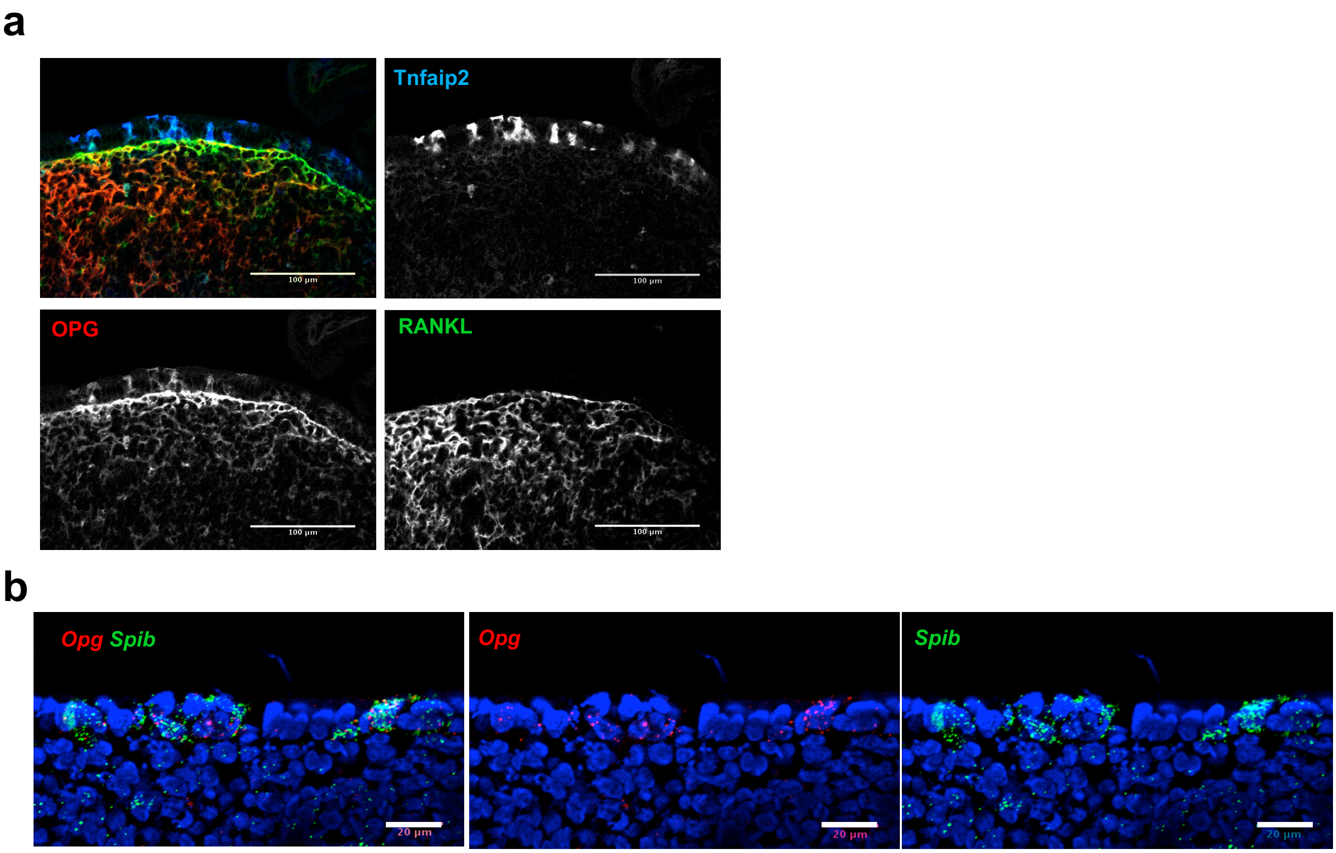

Supplementary Figure 2

OPG released by M cells is distributed throughout the subepithelial dome region.

(**a**) Immunohistochemical staining for RANKL (green), Tnfaip2 (blue), and OPG (red) of a frozen section of a Peyer's patch from a C57BL/6 mouse. OPG protein was detected in both Tnfaip2-positive FAE cells and RANKL-positive subepithelial cells. Each indicated channel is shown separately in grayscale. Scale bars: 100 µm. (**b**) Fluorescence in situ hybridization analysis, with oligonucleotide probes for *Spib* (green) and *Opg* (red), of a Peyer's patch from a C57BL/6 mouse. Each indicated channel is shown separately with cell nuclear staining by DAPI (blue). *Opg* was detected only in the FAE expressing *Spib*. Scale bars: 20 µm.

Supplementary Figure 3

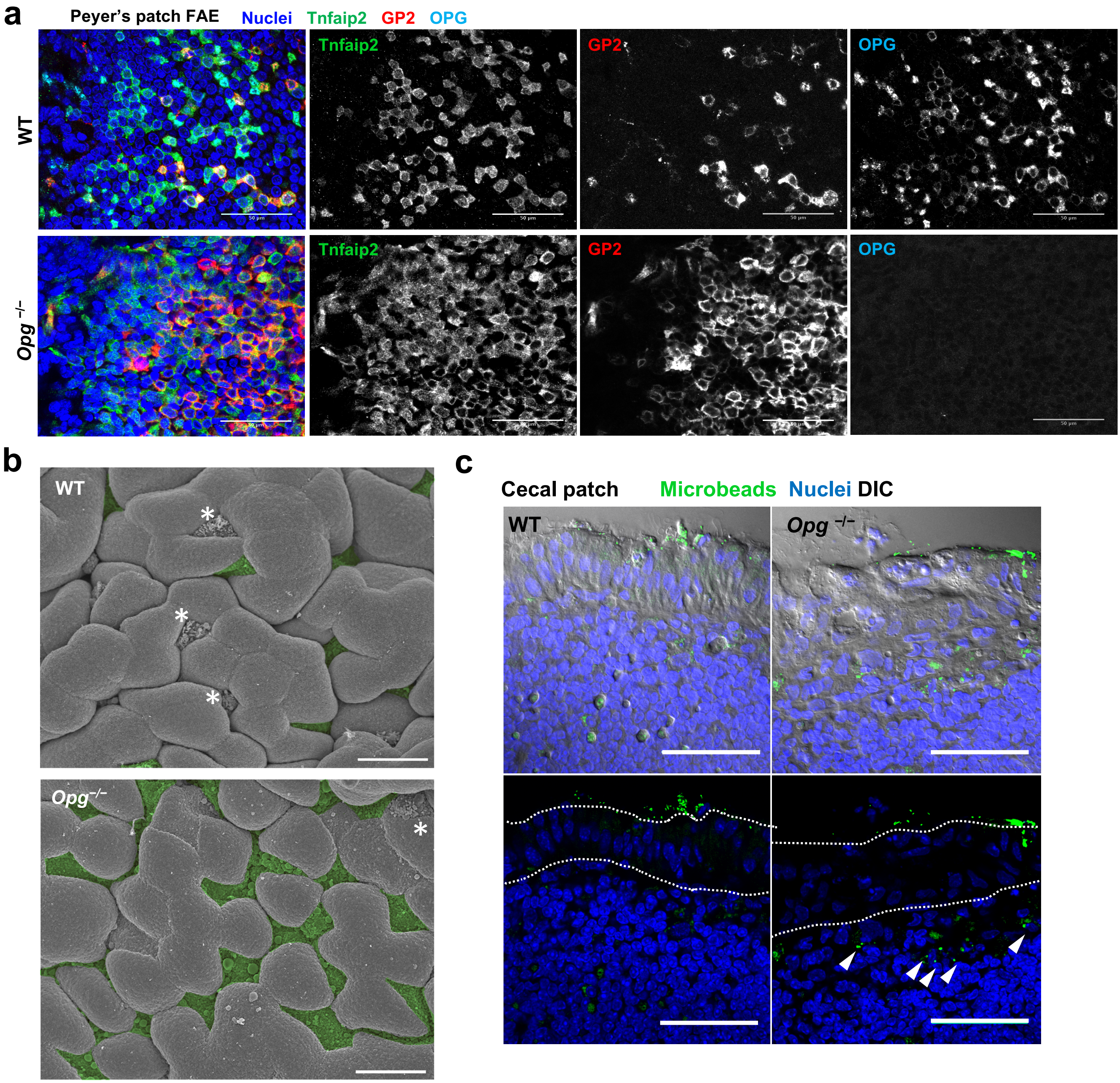

Supplementary Figure 3

M-cell differentiation and activation are enhanced in the absence of *Opg*.

(**a**) High magnification images of whole-mount immunostaining images (shown in Fig. 2a) of Tnfaip2 (green), GP2 (red), OPG (cyan), and cell nuclei (blue) in the FAE of the Peyer's patch from wild-type and *Opg*$^{-/-}$ mice. Each indicated channel is shown separately in grayscale. Scale bars: 50 µm. (**b**) Morphological identification of M cells in Peyer's patches by scanning electron microscopy. M cells (colored green) were identified as cells with short irregular microvilli. Goblet cells (marked with asterisks) were identified as cells with sparse microvilli on their cell surface. Scale bars: 10 µm. (**c**) Latex beads were injected into the ligated intestinal loop of wild-type or *Opg*$^{-/-}$ mice under anesthesia, as described in Methods. Two hours after injection, cecal patches were collected. Cryosections of cecal patches were examined by confocal microscope and the number of fluorescent latex beads were quantified by ImageJ software (shown in Fig. 2D). The areas between two dot lines represent FAE. Arrow heads indicate latex beads. Scale bars: 50 µm.

Supplementary Figure 4

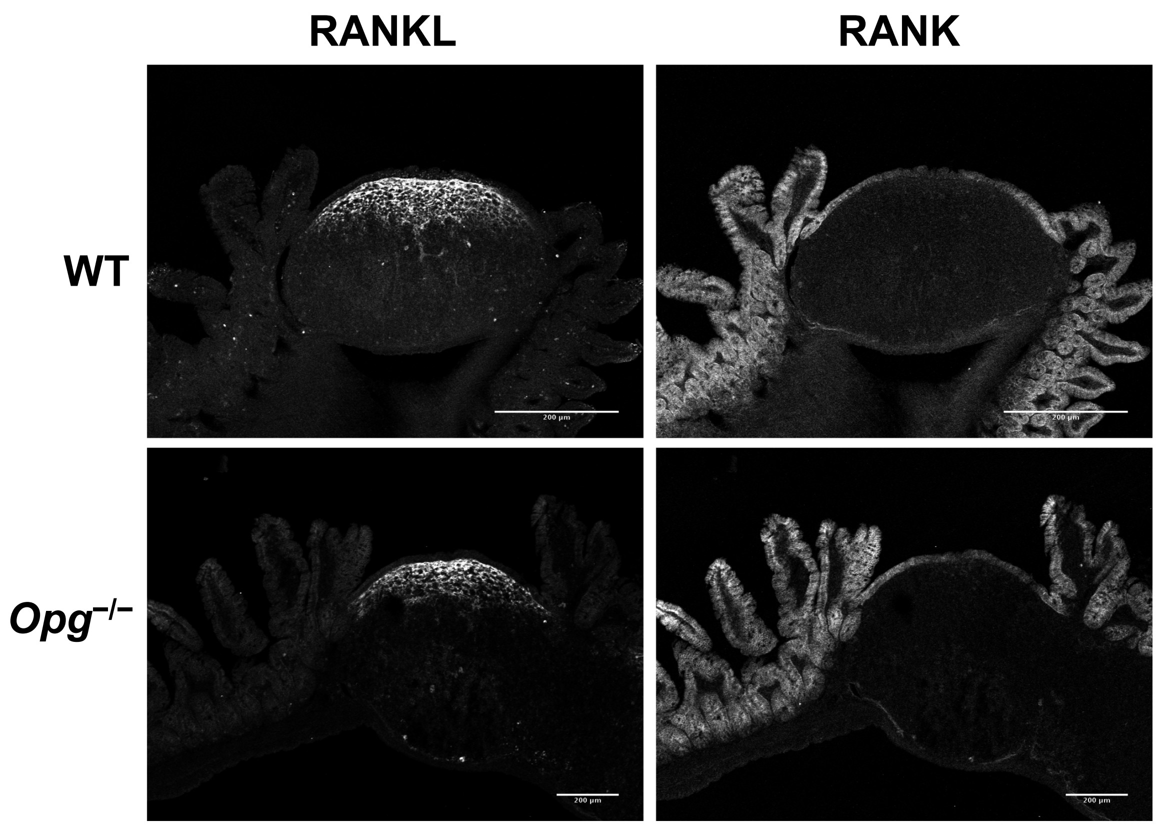

Supplementary Figure 4
RANK and RANKL show similar expression levels and localizations in *Opg*$^{-/-}$ and WT mice.
Immunofluorescence images demonstrate RANK and RANKL antibody staining of Peyer's patches in *Opg*$^{-/-}$ and WT mice. Scale bars: 200 μm.

Supplementary Figure 5

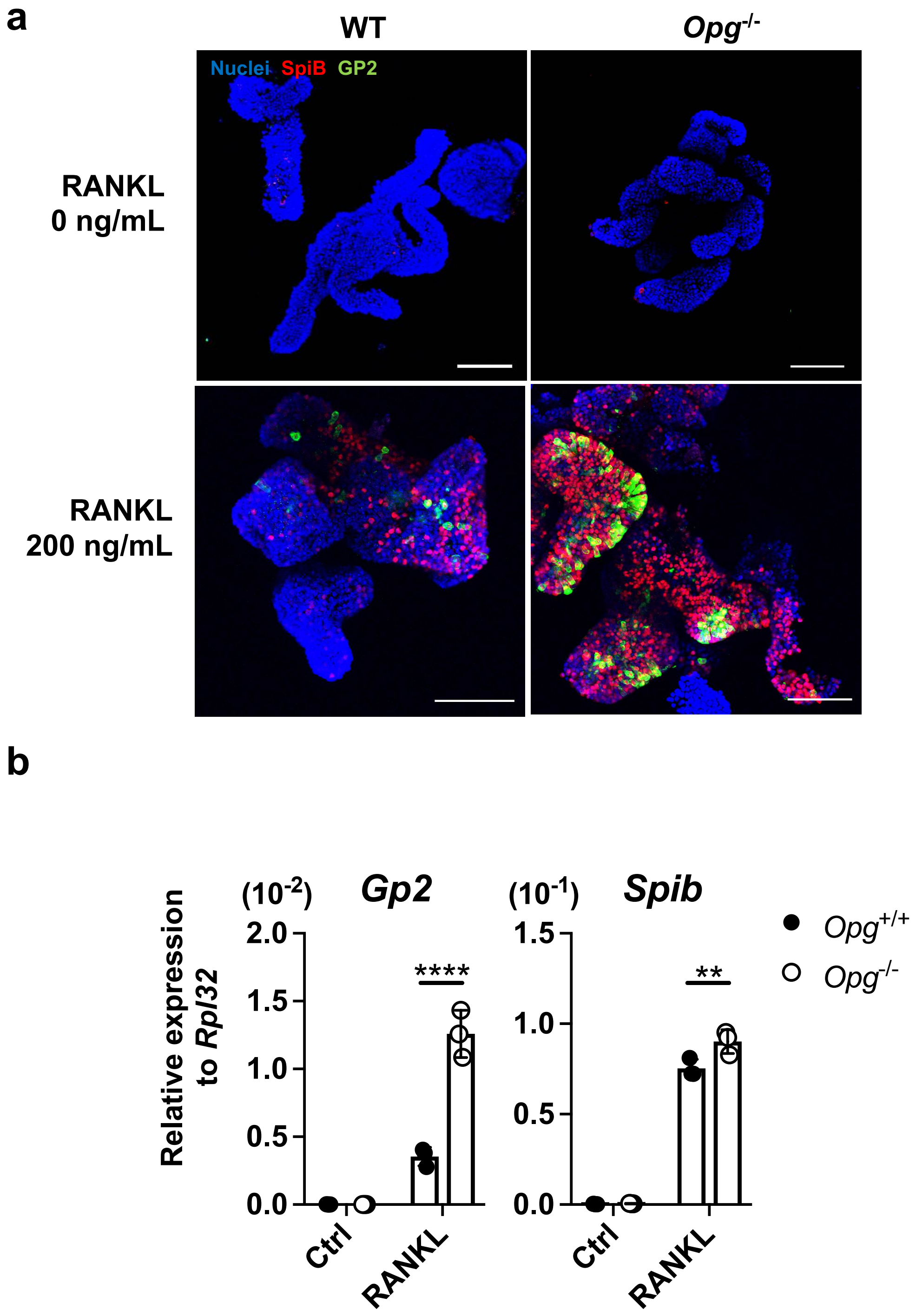

Supplementary Figure 5

Absence of *Opg* enhances M-cell differentiation in organoid culture.

(**a**) Whole-mount-immunohistochemical images of intestinal organoid culture derived from duodenal crypts of indicated mice for Spi-B (red) and GP2 (green). Organoids were cultured with 0 or 100 ng/mL of RANKL for 96 h. Scale bars: 100 µm. (**b**) Quantitative PCR analysis of the expression of *Gp2* and *Spib* in organoid culture. Organoids were cultured with 0 or 100 ng/mL of RANKL for 48 h. Values are presented as the mean ± standard deviation. $^{**}p < 0.01$, $^{****}p < 0.001$, as calculated with Sidak's multiple comparisons test (n = 3 animals). The source data underlying panel **b** are provided as a Source Data file.

Supplementary Figure 6

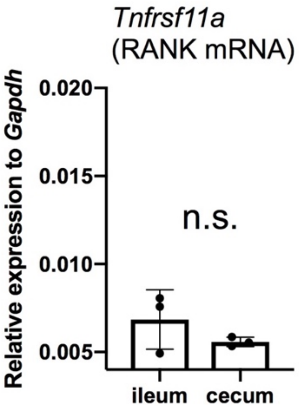

Supplementary Figure 6
Expression levels of RANK mRNA are similar in ileal and cecal epithelia. Epithelia were isolated from ileum or cecum, and data were obtained by quantitative PCR. Data were shown as means ± standard error. n.s.: not significant (Student's t-test, n = 3 animals). The source data are provided as a Source Data file.

Supplementary Figure 7

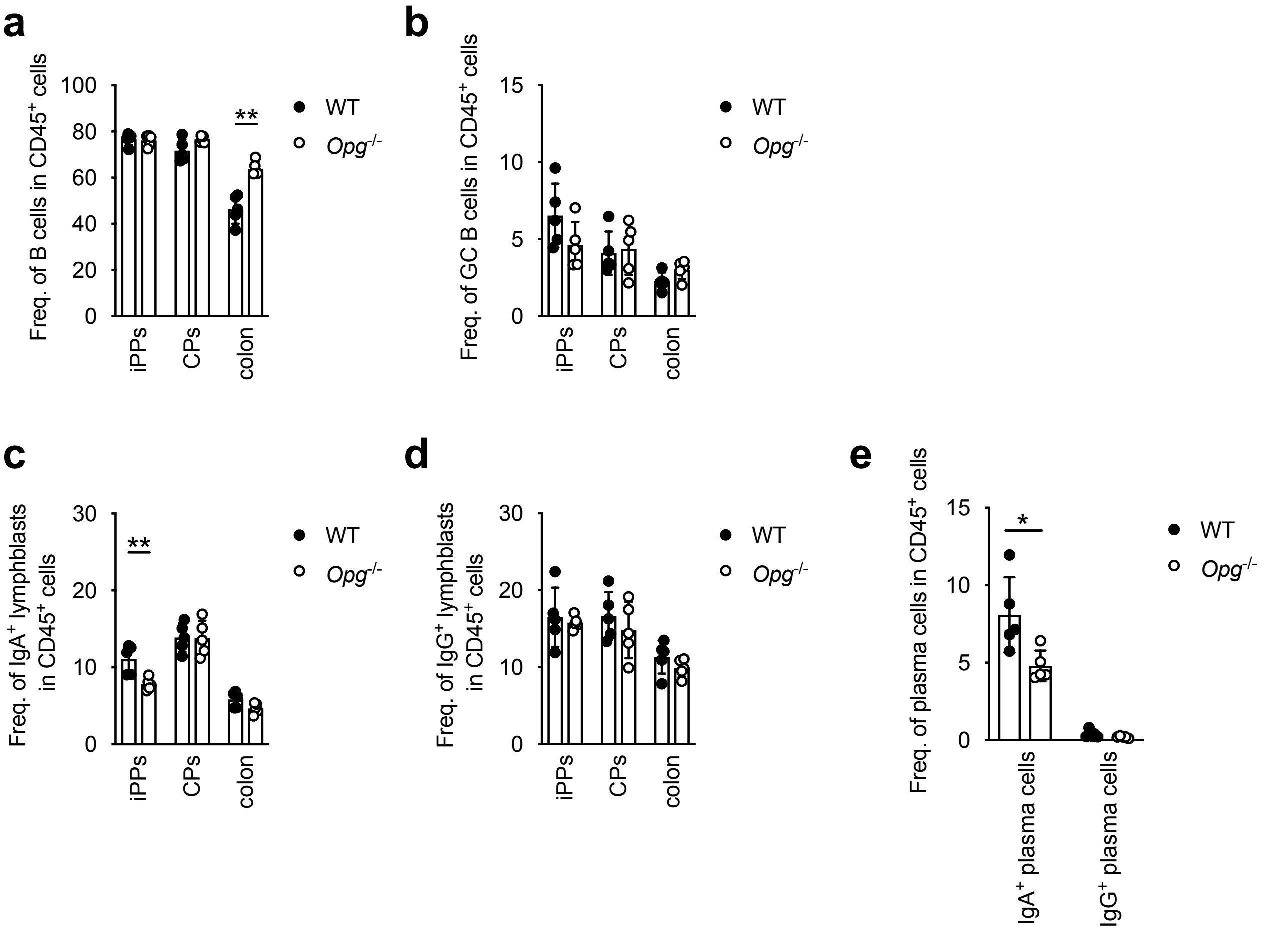

Supplementary Figure 7

Absence of *Opg* promotes mucosal immune responses in the gut, indicated by increased B cell frequencies.

The frequencies of B cells (**a**), germinal center (GC) B cells (**b**), IgA^+ B cells (**c**), IgG^+ B cells (**d**), and plasma cells (**e**) in $CD45^+$ cells in ileal Peyer's patches (iPPs), cecal patches (CPs), and whole colons were quantified by flow cytometry. Data from two independent experiments are presented as the mean ± standard error. $^{**}p < 0.01$, $^{*}p < 0.05$; p-values were calculated with the Student's t-test (n = 5 animals, representative of two independent experiments). This figure is relevant to Figure 5. The source data underlying panel a-**e** are provided as a Source Data file.

Supplementary Figure 8

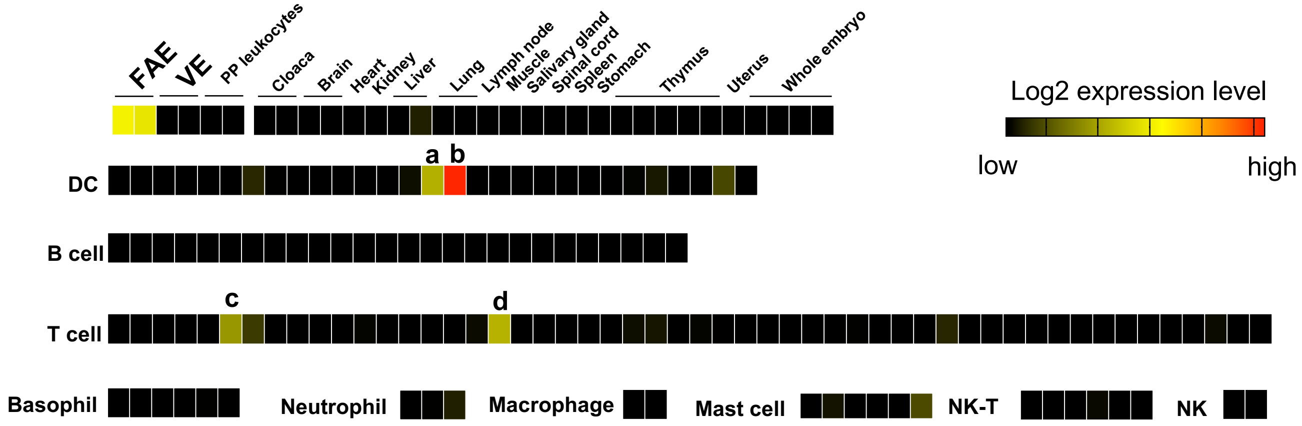

Supplementary Figure 8
OPG mRNA is expressed in the FAE and some immune cell populations
Expression of mouse *Tnfarsf11b* encoding OPG was analyzed using the RefDIC microarray database. Detailed conditions of cell preparation and treatment are available on the RefDIC web site [http://refdic.rcai.riken.jp/welcome.cgi]. FAE: follicle associated epithelium, VE: villus epithelium, DC: dendritic cell, NK-T: natural killer T cell, NK: natural killer cell. **a**: bone marrow-derived DC stimulated with LPS, **b**: *in vitro* cultured DC repeatedly stimulated with antigens, **c**: TCR-stimulated CD4 T cells, **d**: cultured Th1 cells from DO11.10 mice. The source data are provided as a Source Data file.

Supplementary Figure 9

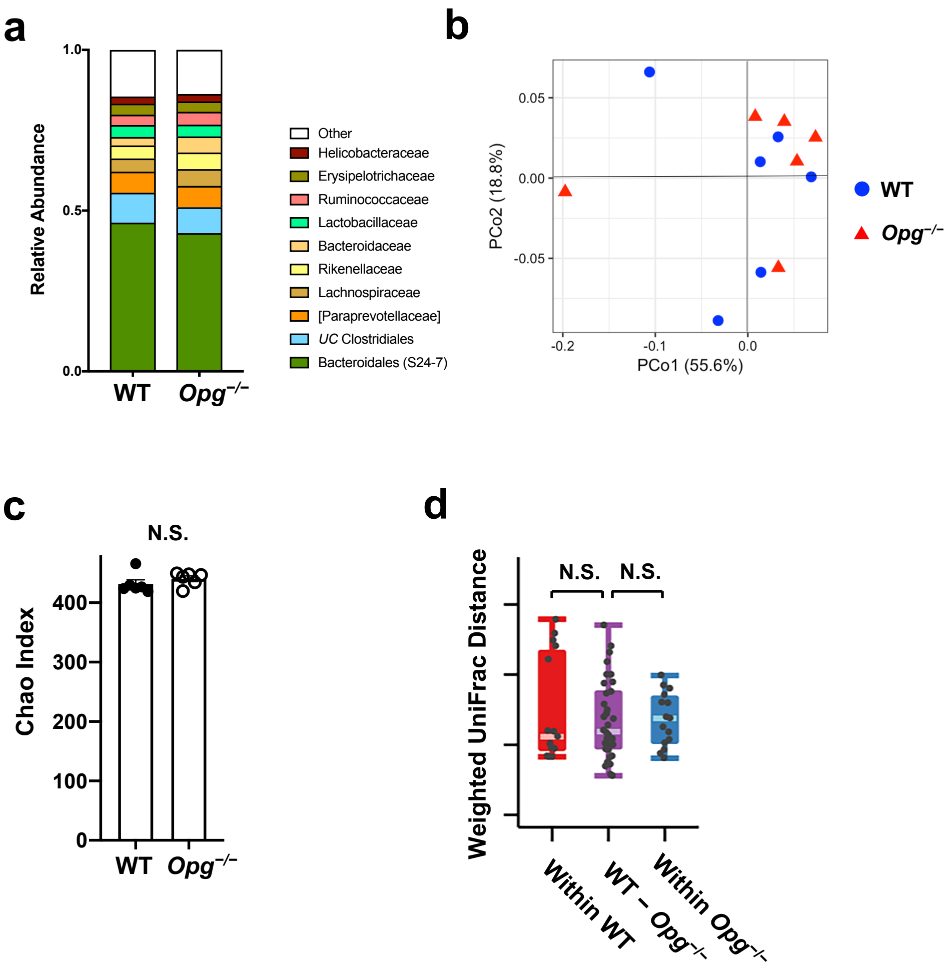

Supplementary Figure 9

Gut microbiota composition and beta diversity are unchanged between WT and *Opg*$^{-/-}$.

(**a**) Relative abundance of the indicated 10 most abundant bacterial families in fecal samples. Families with abundance < 2% were grouped as "other." (**b**) Principal coordinates analysis (PCoA) of weighted UniFrac distances of 16S rRNA genes was illustrated by a scatter plot. Communities from each group of mice did not form a cluster. (**c**) Chao richness index was similar between WT and *Opg*$^{-/-}$ mice. N.S. indicates not significant, as determined by Student's *t*-test, n = 6 animals. Data were shown as means ± standard error. (**d**) The boxplot shows that the weighted UniFrac distances of gut microbiota were similar in WT and *Opg*$^{-/-}$ mice, indicating that diversity did not change between the two groups. N.S. indicates not significant, as determined by Student's t-test, n = 6 animals. The source data underlying panel **a-d** are provided as a Source Data file.

Supplementary Figure 10

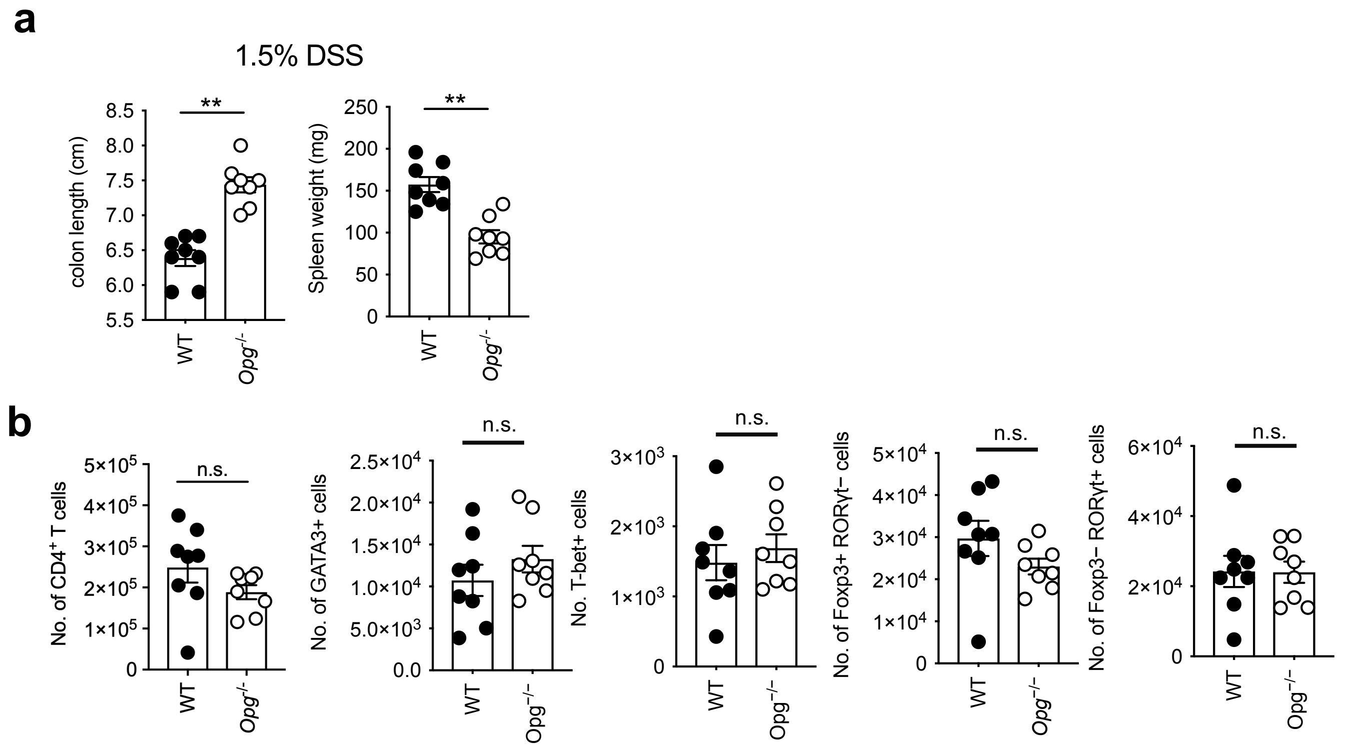

Supplementary Figure 10

Absence of *Opg* has minimal impact on T-cell populations during DSS-induced colitis.

(**a**) Symptoms of DSS-induced colitis were ameliorated in *Opg*$^{-/-}$ mice, as evidenced by the prevention of colonic shortening and splenomegaly, compared with co-housed control mice. Colon and spleen weights were measured after sacrifice at day 9. (**b**) Flow cytometry analysis of indicated immune cells in the colonic lamina propria. B220^{-}CD4^{+}CD3ε^{+} cells were analyzed for Gata3^{+} Th2 cells, T-bet^{+} Th1 cells, RORγT^{-}Foxp3^{+} Treg cells, and RORγT^{+}Foxp3^{-} Th17 cells. Data are shown as mean ± standard deviation and are representative of two independent experiments. $**p < 0.01$; n.s. not significant; *p* values were calculated with the Mann-Whitney U test (n = 8 animals). The source data underlying panel **a** and **b** are provided as a Source Data file.

Supplementary Figure 11

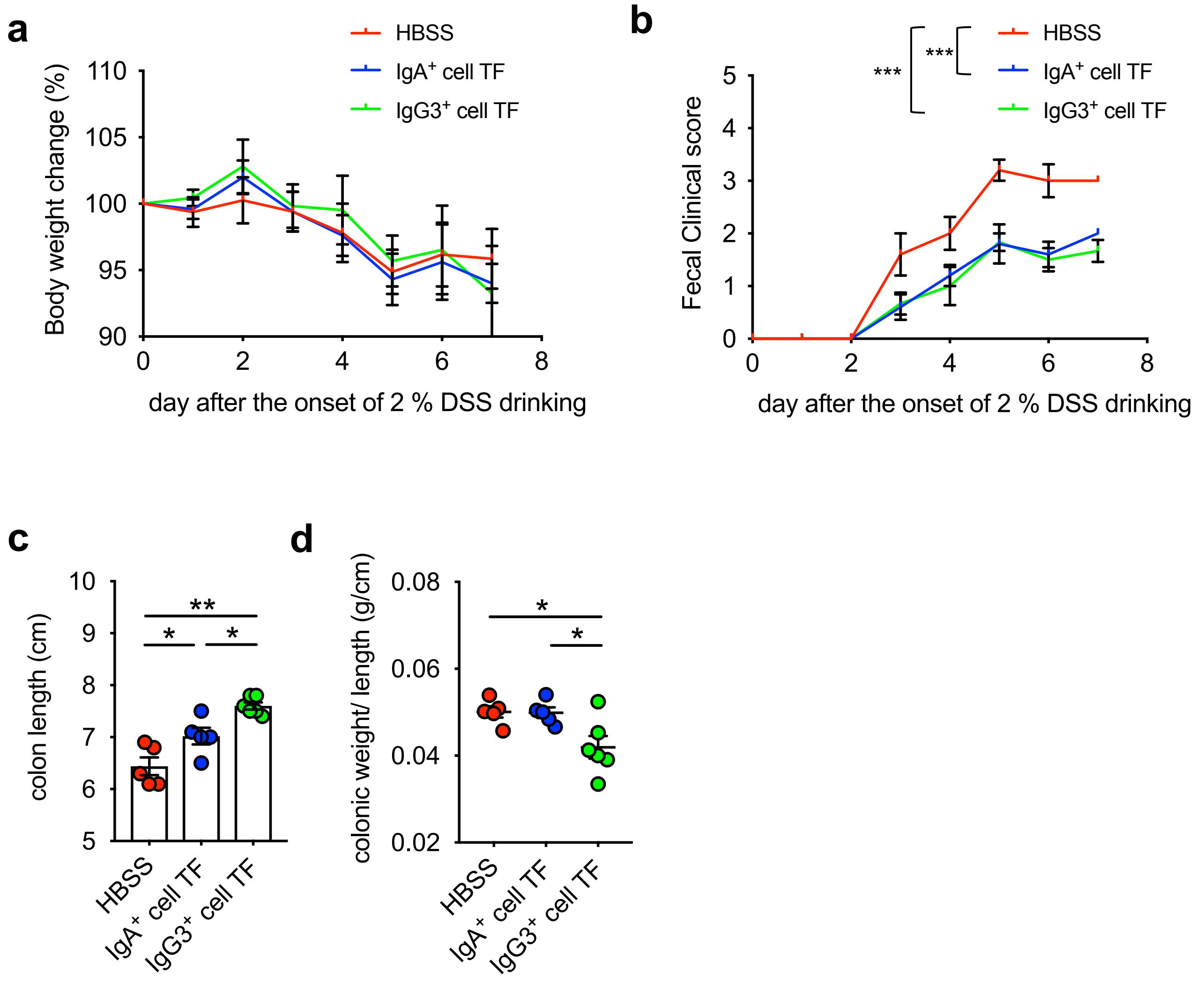

Supplementary Figure 11

Intravenous injection of either IgG3$^+$ or IgA$^+$ cells is protective against DSS colitis.

IgA$^+$ or IgG3$^+$ cells were collected from Peyer's patches, cecal patches, and colonic lamina propria of conventional C57BL/6J mice. These cells were intravenously transferred into *Rag1*$^{-/-}$ mice. After habituation (3 days) to the conventional environment, these mice were administered 2% dextran sodium sulfate (DSS) in drinking water for 7 days. (**a**) Daily changes in body weight during DSS-induced colitis. Changes in body weight percentage were calculated by dividing the body weight on the specified day by the body weight at day 0. (**b**) Stool scores were measured as described in the Methods. (**c**) Colon length was measured after sacrifice at day 8. (**d**) Colon weight per length was measured after sacrifice at day 8. Data from two independent experiments are presented as the mean ± standard deviation. ***p<0.005, **p<0.01, *p < 0.05; one-way ANOVA-test, n = 5 animals (HBSS and IgA$^+$ TF) or 6 animals (IgG3 cell TF) . The source data underlying panel **a-d** are provided as a Source Data file.

Supplementary Figure 12

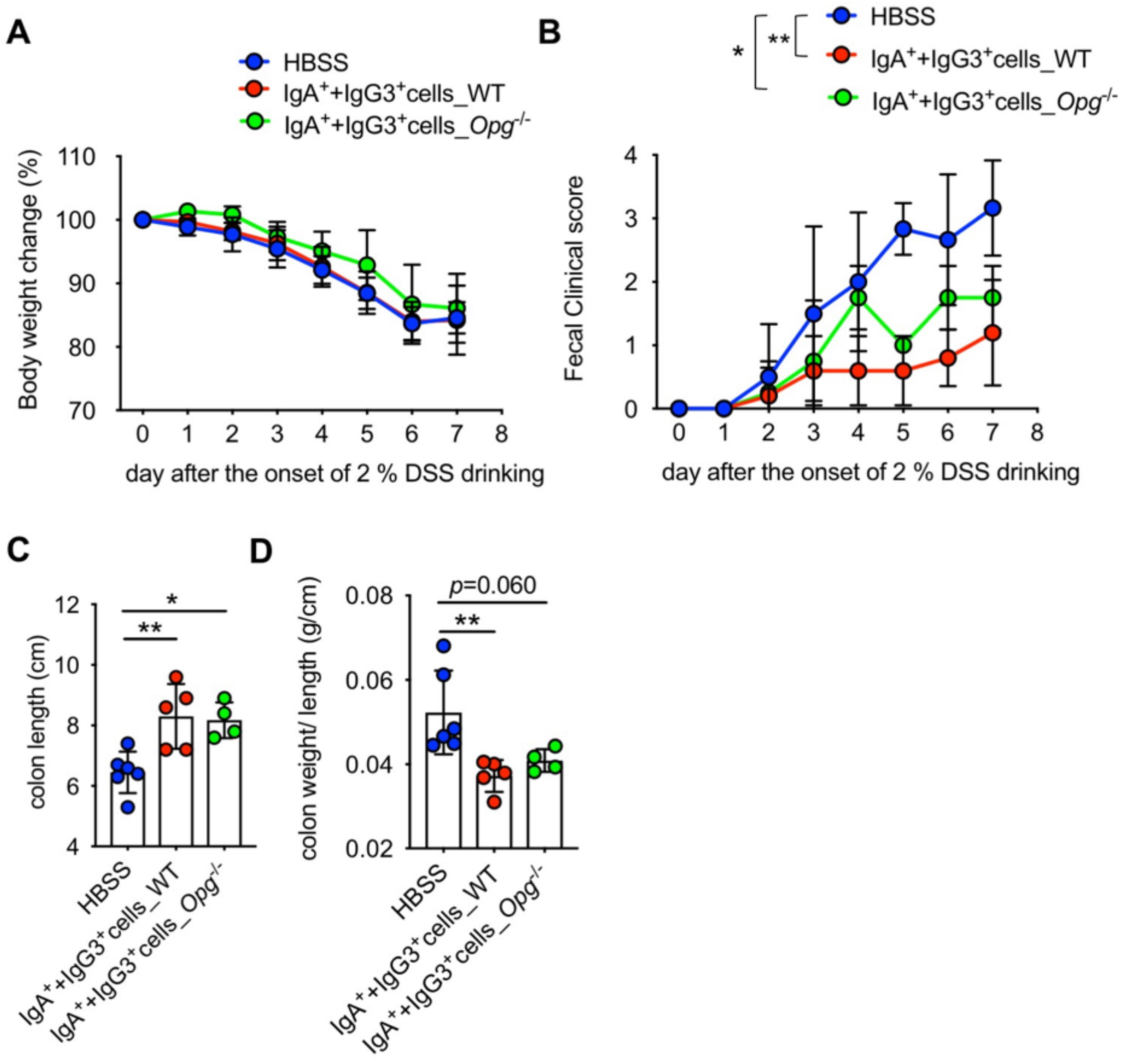

Supplementary Figure 12

Adoptive transfer of IgG3⁺ and IgA⁺ cells from WT and *Opg*$^{-/-}$ mice showed a similar protective effect on the development of DSS-induced colitis.

IgA$^+$ and IgG3$^+$ cells were collected from WT and *Opg*$^{-/-}$ mice. These cells were intravenously transferred into *Rag1*$^{-/-}$ mice. After 3 days, these mice were administered with 2% dextran sodium sulfate (DSS) in drinking water for 7 days. (**a**) Daily changes in body weight during DSS-induced colitis. (**b**) Stool scores were measured as described in the Method section. (**c** and **d**) Colon length and colon thickening were measured on day 8. Data from two independent experiments are shown as the mean ± standard deviation. $^{**}p<0.01$, $^{*}p < 0.05$ (one-way ANOVA-test, $n = 6$ animals). The source data are provided as a Source Data file.

Supplementary Figure 13

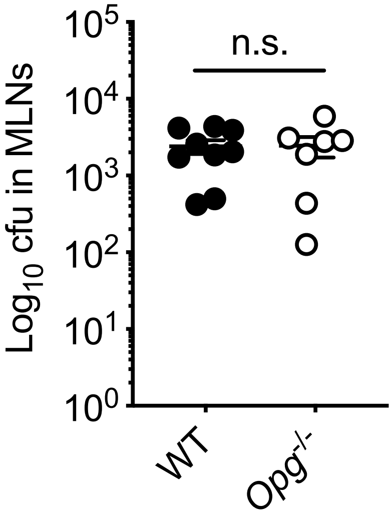

Supplementary Figure 13

Translocation of the *Salmonella* SPI-1 mutant to mesenteric lymph nodes is minimally affected by *Opg* deficiency.

Opg$^{-/-}$ or co-housed WT mice were orally infected with 5×10^7 c.f.u. of *S*. Typhimurium SPI-1 mutant strain. Spleen, liver, Peyer's patches, and mesenteric lymph nodes (MLNs) were collected at 48 h after infection, and the numbers of colonies of the SPI-1 mutant were counted. Colony-forming units (c.f.u.) of mesenteric lymph nodes (MLNs) are shown. No colonies were present in measurements of other tissues. Data from two independent experiments are presented as the mean ± standard error (Unpaired t-test, n = 9 from WT mice and n = 7 from *Opg*$^{-/-}$ mice). The source data are provided as a Source Data file.

Supplementary Figure 14

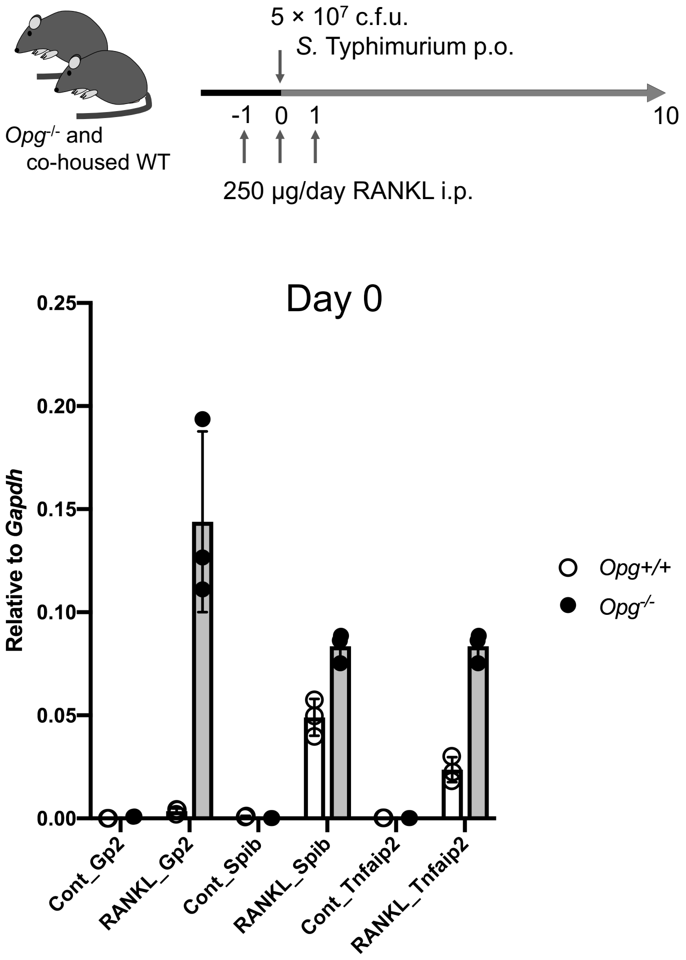

Supplementary Figure 14

Rapid induction of *Gp2* in *Opg*$^{-/-}$ mice by RANKL administration

The expression levels of M-cell associated genes−*Gp2, Spib, Tnfaip2*−were measured by quantitative PCR. mRNA was collected on the second day of the RANKL administration that was the same as salmonella infection (at day 0). Results were represented as relative to *Gapdh* expression. Data are the mean ± standard deviation (*n* = 3 animals). The source data are provided as a Source Data file.

Supplementary Figure 15

a

FCM sorting strategy of Figure 1a

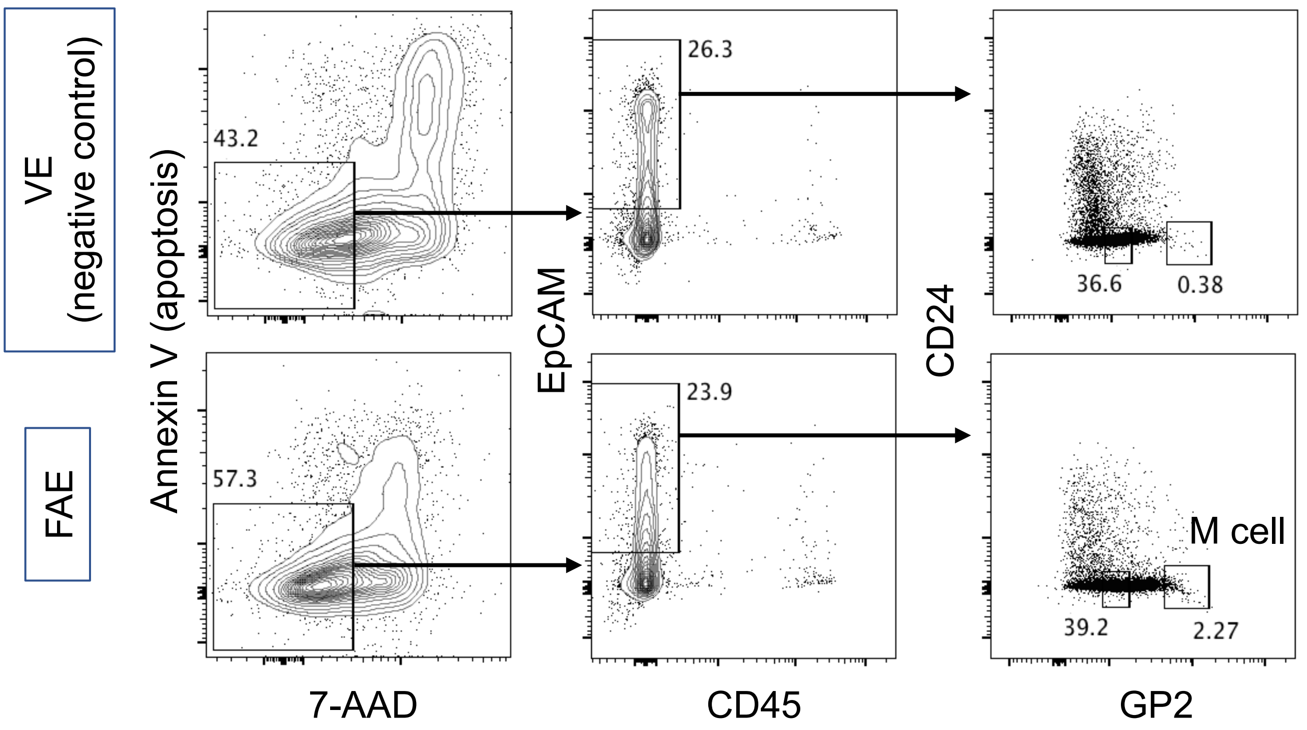

Supplementary Figure 15

b

FCM sorting strategy of Figure 5a-e and Supplementary Figure 7

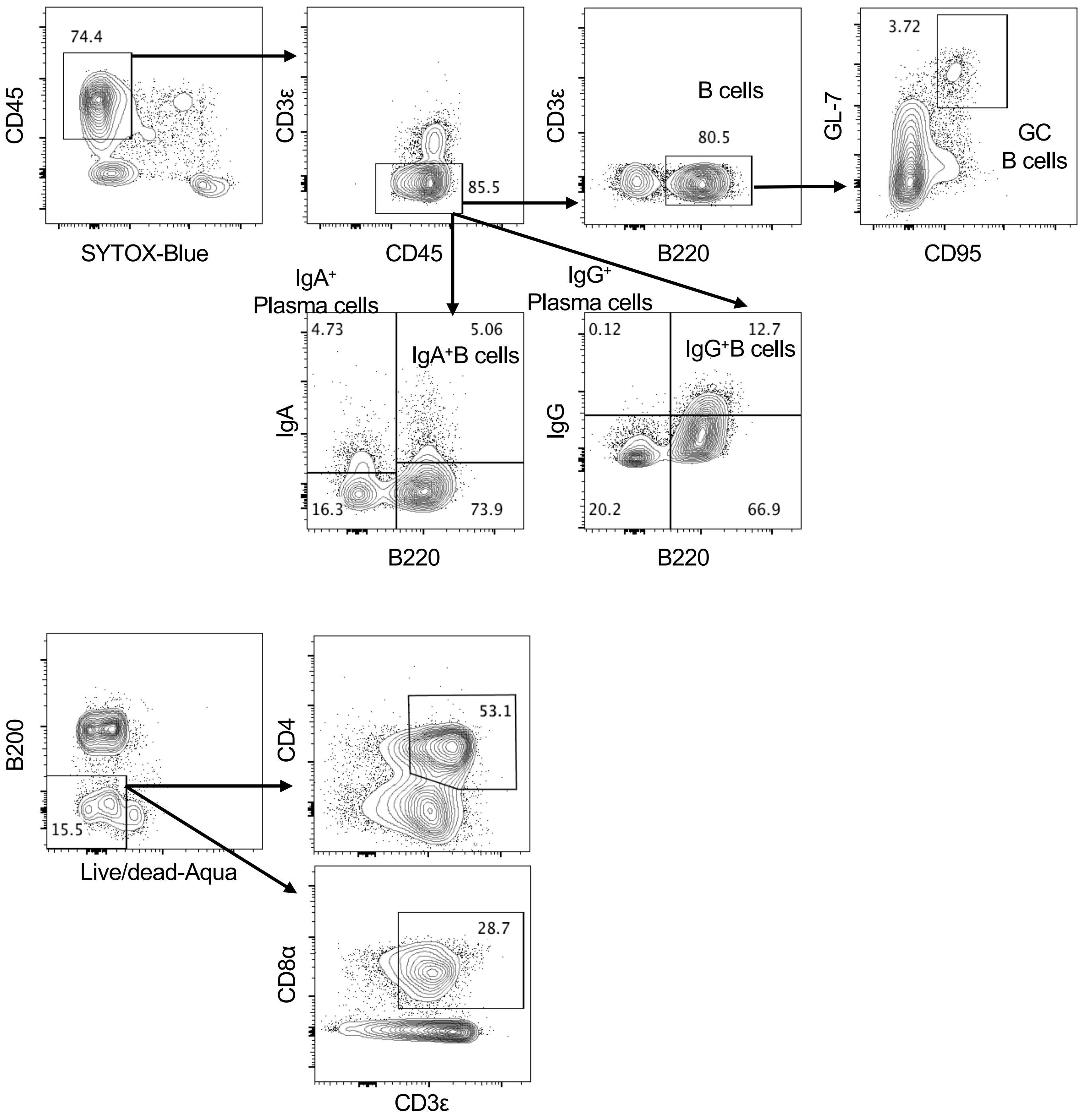

Supplementary Figure 15

c

FCM sorting strategy of Figure 6f

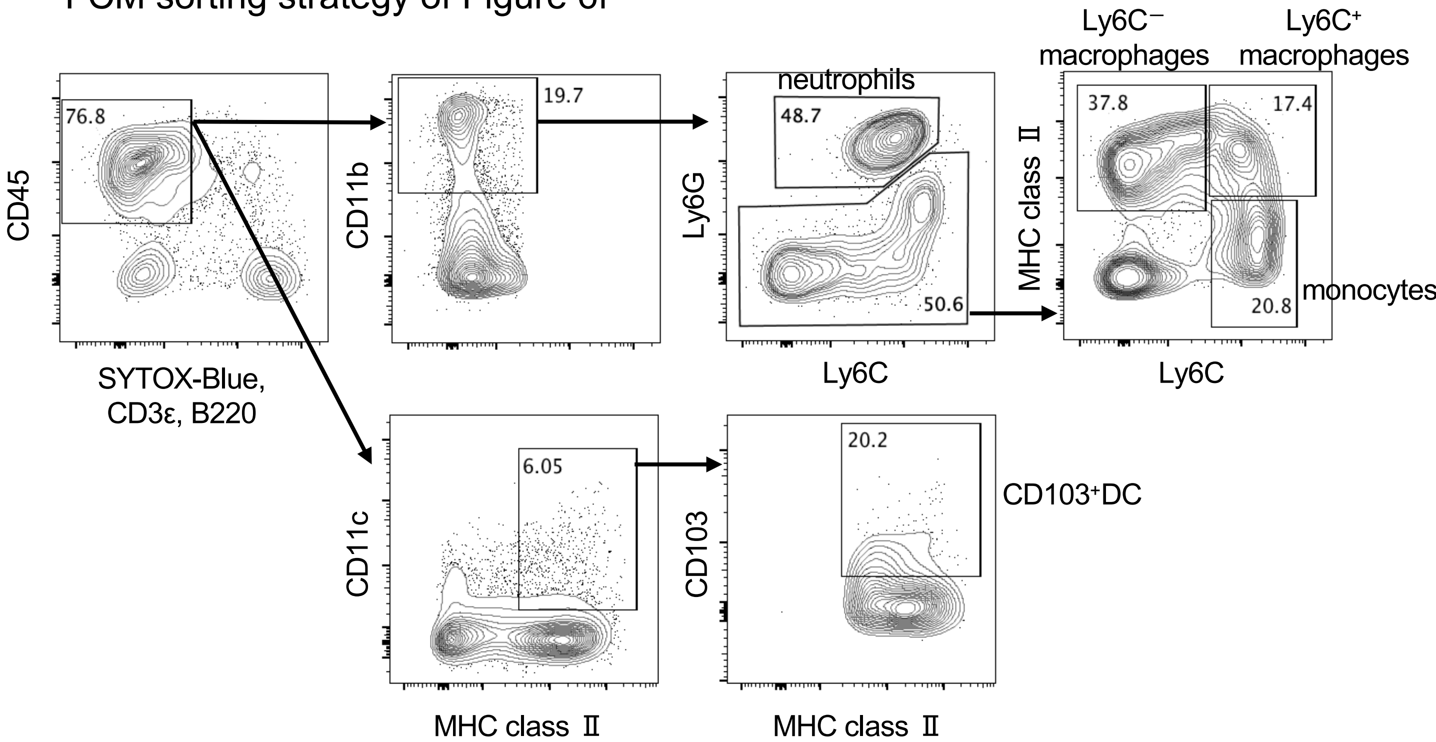

Supplementary Figure 15

d

FCM sorting strategy of Figure 8b, c, e and f

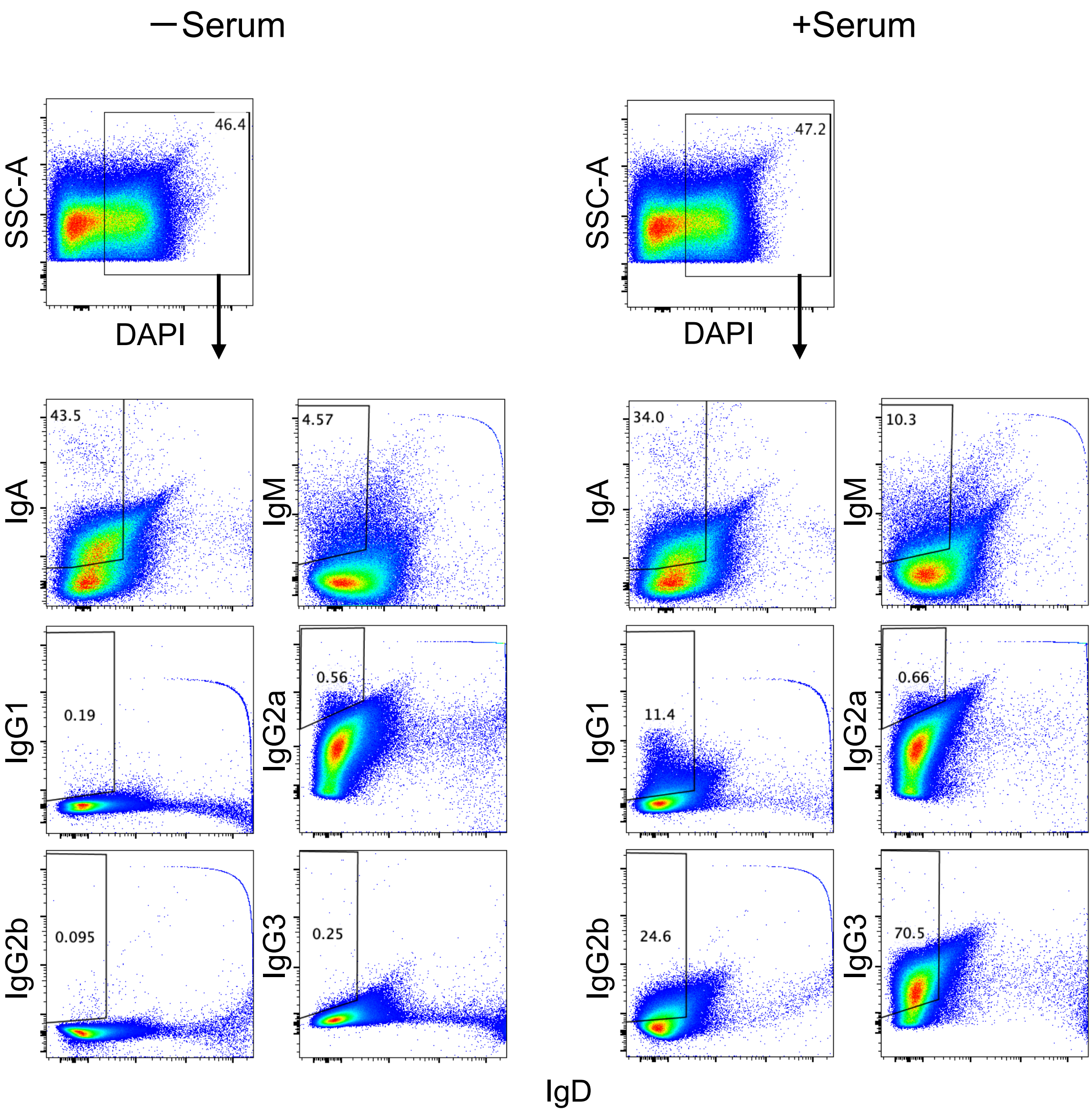

Supplementary Figure 15

e

FCM sorting strategy of Supplementary Figure 10

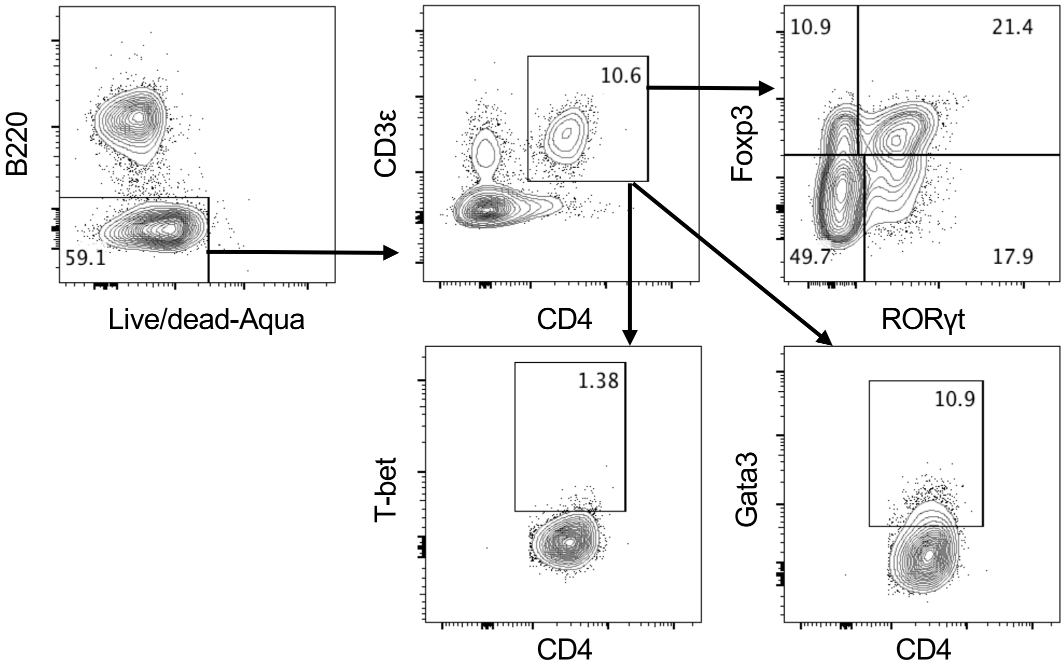

Supplementary Figure 15
FACS sequential gating strategies.
(**a**) A sequential sorting strategy to isolate GP2-positive cells for RNA-seq analysis, which correspond to Figure 1a. (**b**) FACS sorting strategies for subsets of B cells an T cells correspond to Figure 5a-e and Supplementary Figure 7. (**c**) FACS sorting strategies for myeloid lineage cells correspond to Figure 6f. (**d**) FACS sorting strategies for Bug-Ig flow cytometry correspond to Figure 8b, c, e and f.

Supplementary Table 1

List of the oligonucleotide primers

Target gene (Accession No.)	Forward	Reverse
Anxa5 (NM_009673)	ATCCTGAACCTGTTGACATCCC	AGTCGTGAGGGCTTCATCATA
Ccl9 (NM_011338)	CAACAGAGACAAAAGAAGTCCAGAG	CTTGCTGATAAAGATGATGCCC
Ccl20 (NM_001159738)	TGCTCTTCCTTGCTTTGGCATGGGTA	TCTGTGCAGTGATGTGCAGGTGAAGC
Gapdh (NM_008084)	TGTGTCCGTCGTGGATCTGA	TTGCTGTTGAAGTCGCAGGAG
Gp2 (NM_025989)	GATACTGCACAGACCCCTCCA	GCAGTTCCGGTCATTGAGGTA
Marcksl1 (NM_010807)	GGGGCTTATACTCCCAAACC	CCTCCCTCCTGAAGCCTAAC
Spib (NM_019866)	CCGAGGGGAGGGGATCTGAG	GGAGGAGAACTGGAAGACGCCG
Tnfrsf11b (NM_008764)	ACCCAGAAACTGGTCATCAGC	CTGCAATACACACACTCATCACT
Tnfaip2 (NM_009396)	GTGCAGAACCTCTACCCCAATG	TGGAGAATGTCGATGGCCA
Rpl32 (NM_172086.2)	TTCCTGGTCCACAATGTCAA	GGCTTTTCGGTTCTTAGAGGA
16s_V4_515F_Nextera:16s_V4_806R_Nextera	TCGTCGGCAGCGTCAGATGTGTATAAGAGACAGGTGCCAGCMGCCGCGGTAA	GTCTCGTGGGCTCGGAGATGTGTATAAGAGACAGGGACTACHVGGGTWTCTAAT

Supplementary Methods

Mouse small intestinal organoid culture

Small intestinal organoids were derived from WT and *Opg*$^{-/-}$ mice. The duodenum was removed from each mouse and cut into 5-mm segments, which were then incubated in cold PBS with 2 mM EDTA for 5 min and washed by pipetting. The segments were subsequently incubated in cold PBS with 2 mM EDTA for 30 min, and crypts were isolated by pipetting with cold HBSS. Dissociated crypts were strained through 70-μm cell strainers. The crypts were resuspended in DMEM/F12 medium (STEMCELL Technologies, Vancouver, BC, Canada), counted, and resuspended in Matrigel (Corning, Corning, NY, USA). The crypts were plated in 24-well plates with IntestiCult organoid growth medium (STEMCELL Technologies), and the medium was changed every 2 days. For M cell induction, 100 ng/ml or 200 ng/ml recombinant mouse RANKL (Affymetrix, Santa Clara, CA, USA) was added to the medium, after which the cultures were incubated for 2 to 4 days.

Whole-mount immunofluorescence staining of mouse intestinal organoids

Organoids were incubated in cold Gentle Cell Dissociation Reagent (STEMCELL Technologies, Vancouver, BC, Canada) until the Matrigel dissolved, then washed twice with cold PBS, and fixed in 3.7% formaldehyde in PBS for 15 min at room temperature. Fixed organoids were washed three times with PBS and permeabilized with PBS containing 0.2% Saponin and 0.2% bovine serum albumin (BSA) at room temperature for 1 h. Subsequently, the immunohistochemistry process was performed as described above. Images were acquired using a confocal laser microscope FV1000-D-IX81 (Olympus). The primary antibodies used

for wholemount immunostaining are as follows: anti-GP2 antibody (D278-3, MBL, 1:400) and anti-SpiB antibody (AF7204, R&D systems, 1:200).

Metagenomic 16S rRNA sequencing

Approximately 50 mg per fecal sample were collected into a 2-ml tube with 0.1-mm zirconia/silica beads (BioSpec Products, Bartlesville, OK, USA) and 3.0-mm zirconia beads (Bio Medical Sciences, Tokyo, Japan). The fecal samples were homogenized at 1,500 rpm for 10 min with a Shake Master Neo (Bio Medical Sciences) after adding Inhibit EX buffer from the QIAamp Fast DNA Stool Mini Kit (Qiagen). Genomic DNA was subsequently extracted with the kit by following the manufacturer's protocol, then resuspended in 10 mM Tris-HCl buffer at 5 ng/µl. Each library for 16S rRNA genes was prepared in accordance with a protocol from an Illumina technical note, with a modification in the primary amplification PCR step. Briefly, each DNA sample was amplified by PCR with KAPA HiFi HS ReadyMix (KAPA Biosystems) and primers specific for variable region 4 of the 16S ribosomal RNA gene. The primer sequences named 16s_V4_515F_Nextera and 16s_V4_806R_Nextera were listed in the Supplementary Table 2. The PCR products were purified using Agencourt AMPure XP Beads (Beckman Coulter, Brea, CA, USA) and appended by PCR using the Nextera XT index kit (Illumina). The libraries were further purified with Agencourt AMPure XP Beads and pooled after being diluted to 4 nM with 10 mM Tris-HCl buffer. The pooled samples were sequenced using the MiSeq system (Illumina) with a 2 × 300-base pair protocol.

Bacterial composition analysis

The reads were trimmed using Trimmomatic ver. 0.36 with the following parameter: PE TAILING:25 (*1*). The join_paired_ends.py script of QIIME (*2*) was used with the fastq-join method to join paired-end reads, and cutadapt was used to trim sequencing adaptor sequences (*3*). FASTQ files were subsequently converted to FASTA files, and chimera reads were rmoved using the identify_chimeric_seqs.py script and filter_fasta.py script of QIIME with usearch61. The individual sample FASTA files were then concatenated into a single FASTA file, and the pick_open_reference_otus.py script of QIIME was used for operational taxonomic unit (OTU) selection. Taxonomy was assigned using the assign_taxonomy.py script of QIIME with the RDP classifier, and the Greengenes reference database clustered at 97% identity. An OTU table was generated with the make_otu_table.py script of QIIME, and OTUs lower than 0.005% were removed using the filter_otus_from_otu_table.py script of QIIME. Samples were subsampled to a depth of 10,000 reads per sample, and a diversity analysis was performed using the core_diversity_analyses.py script of QIIME.

Supplementary Reference

1. A. M. Bolger, M. Lohse, B. Usadel, Trimmomatic: a flexible trimmer for Illumina sequence data. *Bioinformatics.* **30**, 2114-20 (2014).
2. J. G. Caporaso, J. Kuczynski, J. Stombaugh, K. Bittinger, F. D. Bushman, E. K. Costello, N. Fierer, A. G. Peña, J. K. Goodrich, J.I. Gordon, G. A. Huttley, S. T. Kelley, D. Knights, J. E. Koenig, R. E. Ley, C. A. Lozupone, D. McDonald, B. D. Muegge, M. Pirrung, J. Reeder, J. R. Sevinsky, P. J. Turnbaugh, W. A. Walters, J. Widmann, T. Yatsunenko, J. Zaneveld, R. Knight, QIIME allows analysis of high-throughput community sequencing data. *Nat Methods.* **7**, 335-6 (2010).

3. M. Martin, Cutadapt Removes Adapter Sequences From High-Throughput Sequencing Reads. *EMBnet.Journal.* **17**, 10-12 (2011).
