# Supplementary material for: Osteoprotegerin-dependent M cell self-regulation balances gut infection and immunity
Source: Nat Commun. 2020 Jan 13;11:234. doi: 10.1038/s41467-019-13883-y (PMC6957684; doi:10.1038/s41467-019-13883-y)
Supplement: Supplementary file 3 — Reporting Summary [file 41467_2019_13883_MOESM3_ESM.pdf]

nature research

Corresponding author(s): Koji Hase and Shunsuke Kimura

Last updated by author(s): Nov 26, 2019

Reporting Summary

Nature Research wishes to improve the reproducibility of the work that we publish. This form provides structure for consistency and transparency in reporting. For further information on Nature Research policies, see Authors & Referees and the Editorial Policy Checklist.

Statistics

For all statistical analyses, confirm that the following items are present in the figure legend, table legend, main text, or Methods section.

n/a	Confirmed	
☐	☒	The exact sample size (*n*) for each experimental group/condition, given as a discrete number and unit of measurement
☐	☒	A statement on whether measurements were taken from distinct samples or whether the same sample was measured repeatedly
☐	☒	The statistical test(s) used AND whether they are one- or two-sided<br>*Only common tests should be described solely by name; describe more complex techniques in the Methods section.*
☒	☐	A description of all covariates tested
☐	☒	A description of any assumptions or corrections, such as tests of normality and adjustment for multiple comparisons
☐	☒	A full description of the statistical parameters including central tendency (e.g. means) or other basic estimates (e.g. regression coefficient) AND variation (e.g. standard deviation) or associated estimates of uncertainty (e.g. confidence intervals)
☐	☒	For null hypothesis testing, the test statistic (e.g. *F*, *t*, *r*) with confidence intervals, effect sizes, degrees of freedom and *P* value noted<br>*Give P values as exact values whenever suitable.*
☒	☐	For Bayesian analysis, information on the choice of priors and Markov chain Monte Carlo settings
☒	☐	For hierarchical and complex designs, identification of the appropriate level for tests and full reporting of outcomes
☒	☐	Estimates of effect sizes (e.g. Cohen's *d*, Pearson's *r*), indicating how they were calculated

Our web collection on statistics for biologists contains articles on many of the points above.

Software and code

Policy information about availability of computer code

Data collection	To collecting confocal microscopic data, we used FV10-ASW(Olympus, Tokyo). For flow cytometry, we used BD FACSDiva™ software.
Data analysis	All microscopic data was analyzed by ImageJ Fiji (Schindelin, J.; Arganda-Carreras, I. & Frise, E. et al. Fiji: an open-source platform for biological-image analysis. Nature methods 9(7): 676-682, 2012). Digital RNA-seq were analyzed as described previously (Shiroguchi, K. Jia, T. Z., Sims, P. A. & Xie, X. S. Digital RNA sequencing minimizes sequence-dependent bias and amplification noise with optimized single-molecule barcodes. Proc Natl Acad Sci U S A. 109, 1347-52, 2012).

For manuscripts utilizing custom algorithms or software that are central to the research but not yet described in published literature, software must be made available to editors/reviewers. We strongly encourage code deposition in a community repository (e.g. GitHub). See the Nature Research guidelines for submitting code & software for further information.

Data

Policy information about availability of data

All manuscripts must include a data availability statement. This statement should provide the following information, where applicable:

- Accession codes, unique identifiers, or web links for publicly available datasets
- A list of figures that have associated raw data
- A description of any restrictions on data availability

Digital RNA sequencing data sets have been deposited in the Genome Expression Omnibus database under the accession number “GSE108529 [https://www.ncbi.nlm.nih.gov/geo/query/acc.cgi?acc=GSE108529]”. Metagenomic 16S rRNA sequencing data have been deposited in the DNA Data Bank Japan (DDBJ) under the accession number “DRP004759 [https://ddbj.nig.ac.jp/DRASearch/study?acc=DRP004759]”. The source data underlying Figs 1c, g, 2b, c, e, f, 3b, c, 4b-d, 5a-g, 6a, b, d-f, 7a-h, 8b, c, e, f, 9a, c, and 9d and Supplementary Figs 5b, 6, 7a-e, 8, 9a-d, 10a, b, 11a-d, and 12 are provided as a Source Data file. The dataset generated and analyzed during the current study are available from the corresponding authors upon reasonable request.

Field-specific reporting

Please select the one below that is the best fit for your research. If you are not sure, read the appropriate sections before making your selection.

☒ Life sciences ☐ Behavioural & social sciences ☐ Ecological, evolutionary & environmental sciences

For a reference copy of the document with all sections, see nature.com/documents/nr-reporting-summary-flat.pdf

Life sciences study design

All studies must disclose on these points even when the disclosure is negative.

Sample size	We did not performed sample-size calculation. The sample size was empirically started at 3 or 5, and was increased at the next experiment when there was no significant difference. Ultimately at least two independent experiments were conducted to draw conclusions.
Data exclusions	No data was excluded in all our experiments.
Replication	We confirmed that all attempts at replication were successful.
Randomization	All samples in this experiments were randomly allocated.
Blinding	We were not blinded to group allocation during data collection and analysis. Because the data collection and analysis were performed with quantitative instruments to maintaining objectivity.

Reporting for specific materials, systems and methods

We require information from authors about some types of materials, experimental systems and methods used in many studies. Here, indicate whether each material, system or method listed is relevant to your study. If you are not sure if a list item applies to your research, read the appropriate section before selecting a response.

Materials & experimental systems

n/a	Involved in the study	
☐	☒	Antibodies
☒	☐	Eukaryotic cell lines
☒	☐	Palaeontology
☐	☒	Animals and other organisms
☒	☐	Human research participants
☒	☐	Clinical data

Methods

n/a	Involved in the study	
☒	☐	ChIP-seq
☐	☒	Flow cytometry
☒	☐	MRI-based neuroimaging

Antibodies

Antibodies used	The primary antibodies used for immunofluorescence staining are as follows: anti-OPG antibody (catalog number AF549, R&D systems, 1:400): anti-GP2 antibody (D278-3, MBL, 1:400): anti-Tnfaip2 antibody (Kimura et al., ref. 14, 1:200): anti-SpiB antibody (AF7204, R&D systems, 1:200): anti-RelB antibody (sc-226, Santa Cruz Biotechnology, 1:100): and anti-RANKL antibody (14-5952, eBioscience, 1:100).<br>The primary antibodies used for western blotting are as follows: anti-RelB antibody (catalog number: sc-226, Santa Cruz Biotechnology, dilution 1:2000); anti-p100/p52 antibody (#4882, Cell Signaling Technology, 1:1000); anti-Lamin A/C antibody (GTX1116777, GeneTex, 1:2000); and anti-Rpt4 antibody (BML-PW8830, Enzo Life Science, 1:2000).<br>The antibodies used for flow cytometry are as follows: redFluor710 anti-mouse CD3ε antibody (catalog number 80-0032, Tonbo Biosciences, dilution 1:800); eFluor450 anti-mouse CD3ε antibody (48-0031-80, Thermo Fisher Scientific, 1:800); APC-H7 anti-mouse CD4 antibody (560246, BD Bioscience, 1:800); PE anti-mouse CD8a antibody (12-0081-81, Thermo Fisher Scientific, 1:800); PerCP-Cyanine5.5 anti-CD11b (45-0112-80, Thermo Fisher Scientific, 1:800); PE-Cy7 anti-mouse CD11c antibody (117318, BioLegend, 1:800); APC anti-mouse CD24 antibody (101814, BioLegend, 1:400); PE anti-mouse CD45 antibody (103105, BioLegend, 1:800); FITC anti-mouse CD45.1 antibody (35-0453, Tonbo Bioscience, 1:800); APC-eFluor 780 anti-mouse CD45.2 antibody (47-0454-82, Thermo Fisher Scientific, 1:800); APC-Cy7 anti-mouse CD45R (552094, BD Bioscience, 1:800); FITC anti-mouse CD95 antibody (561979, BD Bioscience, 1:400); APC anti-mouse CD103 antibody (121414, BioLegend, 1:400); PE anti-mouse CD326 antibody (118206, BioLegend, 1:400); eFluor 660 anti-GL7 antibody (50-5902-80, Thermo Fisher Scientific, 1:400); Alexa488 anti-GP2 antibody (D278-A48, MBL, 1:800); Alexa488 anti-FoxP3 antibody (53-5773-80, Thermo Fisher Scientific, 1:100); eFluor 660 anti-Gata3 antibody (50-9966-41, Thermo Fisher Scientific, 1:100); Alexa488 anti-mouse Ly6G/Ly-6C antibody (108419, BioLegend, 1:400); APC anti-Ly6C antibody (560595, BD Bioscience, 1:400); APC-Cy7 anti-mouse Ly-6G antibody (560600, BD Bioscience, 1:400); biotin anti-IgA antibody (556978, BD Bioscience, 1:800); FITC anti-IgG antibody (406001, BioLegend, 1:800); PerCP-Cy5.5 anti- IFN-γ antibody (45-7311-80, Thermo Fisher Scientific, 1:400); PE anti-IL-17A antibody (506903, BioLegend, 1:400); eFluor450 anti-MHC class II antibody (48-5321-80, Thermo Fisher Scientific, 1:800); PE anti-ROR-γt antibody (562607, BD Bioscience, 1:100); and PerCP-Cy5.5 anti-T-bet antibody (45-5825-82, Thermo Fisher Scientific, 1:100).

Validation	The specificity of anti-Tnfaip2 antibody was confirmed by Western blotting and immunohistochemistry of tissues from Tnfaip2 knockout mice. Likewise, The specificity of anti-OPG antibody was confirmed by immunohistochemistry of tissues from OPG knockout mice. All other antibodies used in this study were validated by manufactures.

Animals and other organisms

Policy information about studies involving animals; ARRIVE guidelines recommended for reporting animal research

Laboratory animals	Opg–/– mice on a C57BL/6J background were generated as previously described (Mizuno, A. et al. Severe osteoporosis in mice lacking osteoclastogenesis inhibitory factor/osteoprotegerin. Biochem Biophys Res Commun. 247, 610-5, 1998). C57BL/6J mice were purchased from CLEA Japan, Inc. (Tokyo, Japan) or Japan SLC, Inc. (Shizuoka, Japan).
Wild animals	This study did not involve wild animals.
Field-collected samples	This study did not involve samples collected from the field.
Ethics oversight	Protocols approved by Hokkaido University, IMSUT, and Keio University were used for all animal experiments.

Note that full information on the approval of the study protocol must also be provided in the manuscript.

Flow Cytometry

Plots

Confirm that:

☒ The axis labels state the marker and fluorochrome used (e.g. CD4-FITC).

☒ The axis scales are clearly visible. Include numbers along axes only for bottom left plot of group (a 'group' is an analysis of identical markers).

☒ All plots are contour plots with outliers or pseudocolor plots.

☒ A numerical value for number of cells or percentage (with statistics) is provided.

Methodology

Sample preparation	Single cell suspensions of gut tissues were prepared as described previously40. Briefly, colons, distal Peyer's patches (ileal Peyer's patches), and cecal patches were dissociated with RPMI1640 medium containing 12.5 mM HEPES (pH7.4), 2% fetal bovine serum, 0.5 mg/ml collagenase (Wako Pure Chemical Industries), and 0.5 mg/ml DNase I (Wako Pure Chemical Industries) at 37 °C for 30 min. Cells were isolated by Percoll gradient separation.<br>Isolated cells were incubated with anti-CD16/CD32 (FcγR) (2.4G2) antibody to block non-specific reactions and subsequently stained by specific antibody. For intracellular cytokine staining, isolated cells were cultured with phorbol 12-myristate 13-acetate, ionomycine, and brefeldin A for 4.5 h prior to cell surface staining. After surface staining, dead cells were stained by Live/Dead Aqua. Cells were fixed by BD Cytofix/CytoPerm (BD Biosciences) for 15 min. After fixation, cells were stained by various antibodies directed against intracellular cytokines.<br>For Bug-Ig flow cytometry, feces containing bacteria were washed with sterile PBS and resuspended with sterile PBS containing 1% BSA (1% BSA/PBS). The washed bacteria were mixed with or without serum diluted 1:25 in 1% BSA/PBS. After being washed with 1% BSA/PBS, bacteria were stained using primary antibodies.
Instrument	LSRII and FACS Aria (BD Biosciences)
Software	Data were collected and analyzed by BD FACSDiva™ software.
Cell population abundance	In this study, cell sorting was not performed.
Gating strategy	For detection of lymphoid cell populations, Lymphocyte, monocyte and neutrophil populations were gated on a forward scatter (FSC)/side scatter (SSC) plot. Dead cells were excluded as SYTOX-blue positive cells in Fig. 4, Fig. 5F or Live Dead Aqua-V500 negative cells in Fig. 5. Detailed gating strategies were shown as below. Positive and negative were discriminated for each cell population by counter plot (contour line plot). In the case where no independent cell population was formed, the wide interval between contour lines was defined as boundaries between positive and negative.<br>For Bug-Ig flow cytometry, bactreria were gated on DAPI-positive/SSC-A plot. For determining boundaries between "positive" and "negative", unstained bacteria were used as negative controls.<br><br>Figure 5 and Supplementary Figure 7<br>initial gatings: SYTOX-blue-negative CD45-PE-positive → CD3-APC-negative CD45-PE-positive<br>a: CD3-APC-negative B220-APCCy7-positive<br>b: CD95-Alexa700-positive GL-7-V421-positive<br>c: IgA-PECy7-positive B220-APCCy7-positive<br>IgG-Alexa488-positive B220-APCCy7-positive<br>d: IgA-PECy7-positive B220-APCCy7-negative<br>IgG-Alexa488-positive B220-APCCy7-negative<br>e: Live Dead Aqua-V500-negative B220-V421-negative → CD3-Alexa700-negative CD4-APCH7-positive<br>Live Dead Aqua-V500-negative B220-V421-negative → CD3-Alexa700-negative CD8a-Alexa488-positive

Figure 6f
CD45+ CD3e-B220- cells: (SYTOX-blue+ CD3+B220)-V500-negative CD45-Alexa700-positive
This is used as an initial gate for following figures.
monocytes: CD11b-PerCPCy5.5-positive → Ly6G-APC-Cy7-negative Ly6C-PE-Cy7+/- → MHC-classII-V421-negative Ly6C-PE-Cy7-positive
Ly6C+ macrophage: CD11b-PerCPCy5.5-positive → Ly6G-APC-Cy7-negative Ly6C-PE-Cy7+/- → MHC-classII-V421-positive Ly6C-PE-Cy7-positive
Ly6c- macrophage: CD11b-PerCPCy5.5-positive → Ly6G-APC-Cy7-negative Ly6C-PE-Cy7+/- → MHC-classII-V421-positive Ly6C-PE-Cy7-negative
CD103+ DC: MHC-classII-V421-positive CD11c-PE-positive → MHC-clasII-V421-positive CD103-APC-positive
neutrophils: CD11b-PerCPCy5.5-positive → Ly6G-APC-Cy7-positive Ly6C-PE-Cy7+/-

Figure 8
The initial gate: DAPI-positive SSC-A
IgA+: IgD-PE CF594-negative IgA-APC-positive
IgM+: IgD-PE CF594-negative IgM-PE-positive
IgG1+: IgD-PE CF594-negative IgG1-PECy7-positive
IgG2a+: IgD-PE CF594-negative IgG2a-PerCPCy5.5-positive
IgG2b+: IgD-PE CF594-negative IgG2b-PE-positive
IgG3+: IgD-PE CF594-negative IgG3-Alexa488-positive

☒ Tick this box to confirm that a figure exemplifying the gating strategy is provided in the Supplementary Information.
