# Supplementary material for: Osteoprotegerin-dependent M cell self-regulation balances gut infection and immunity
Source: Nat Commun. 2020 Jan 13;11:234. doi: 10.1038/s41467-019-13883-y (PMC6957684; doi:10.1038/s41467-019-13883-y)
Supplement: Supplementary file 4 — Description of Additional Supplementary Files [file 41467_2019_13883_MOESM4_ESM.pdf]

Description of an Additional Supplementary File

File Name: Supplementary Data 1

Description: Digital RNA-seq data a for comparative analysis of $GP2^+$ and $GP2^−$ cells. Genes were arranged in descending order of expression in $GP2^+$ cells.
